# Exploration of body weight in 115 000 young adult dogs of 72 breeds

**DOI:** 10.1038/s41598-022-27055-4

**Published:** 2023-01-09

**Authors:** L. Andersson, U. Emanuelson, S. Ringmark, C. R. Bjørnvad, Å. Hedhammar, K. Höglund

**Affiliations:** 1 Department of Anatomy, Physiology and Biochemistry, Swedish University of Agricultural Sciences, Box 7054, 75007 Uppsala, Sweden; 2 Department of Clinical Sciences, Swedish University of Agricultural Sciences, Box 7054, 75007 Uppsala, Sweden; 3 Department of Veterinary Clinical Sciences, Faculty of Health and Medical Sciences, University of Copenhagen, Dyrlægevej 16, 1870 Frederiksberg C, Denmark

**Keywords:** Physiology, Epidemiology

## Abstract

High body weight (BW), due to large size or excess body fat, has been associated with developmental and metabolic alterations, and degenerative diseases in dogs. Study objectives were to determine mean BW in young adult dogs of different breeds, including changes over a 10-year period. Body weight data from the official Swedish hip dysplasia screening program were used, including data from dogs screened at 1–2.5 years of age, in breeds with ≥ 15 individual observations/year during 2007–2016. Mean BW per breed and sex was established from 114 568 dogs representing 72 breeds. Estimates of breed BW showed significant change in 33 (45%) breeds over the 10-year period. Body weight increased in five breeds (2–14% change) and decreased in 26 breeds (1–8% change). In two breeds, BW increased in male and decreased in female dogs. This observational study provides extensive breed BW data on young adult dogs. The change in breed BW, noted in almost half of the breeds, could be due to changes either in size or in body fat mass. In certain breeds, the change in BW over time might have an impact on overall health. Studies with simultaneous evaluation of BW and body condition over time are warranted.

## Introduction

Dogs show unique diversity in morphology and body size, with small to giant sized breeds^1,2^. Hence, the variation in body weight (BW) between breeds is extensive, and at the same time BW can also vary considerably within breeds^3^.

The adult BW of a dog is reached at different ages between breeds and depends on both genetic and environmental factors^2–5^. High BW in dogs, due to large size or excess amount of body fat, is a risk factor for several developmental and metabolic alterations^6–9^ and degenerative joint diseases, such as hip dysplasia and osteoarthritis, and can negatively affect quality of life and lifespan^10–16^. High BW due to excess body fat is an escalating problem in dogs as well as in humans^17^, and studies have reported 20–59% of dogs being overweight or obese^18–25^.

The basis for indicated breed BWs in breed standards are often unknown. Given the connection between BW and canine health, scientifically evaluated breed BWs of young adult dogs of different breeds are warranted. In addition, it is currently unknown whether breed BW of young adult dogs has been stable over time or been subjected to change.

In the official screening program for hip dysplasia, run by the Swedish Kennel Club (SKC), recording of the dog’s BW at time of screening has been required since 2005. The program is available for all breeds for an official screening result from an age of 12 months (or 18 months for certain large breeds) and with mandatory registration and publication of all results in the SKC database. With approximately 70% of dogs in Sweden being registered in SKC, the SKC database holds an extensive amount of individual observations on BW in young adult dogs of different breeds^26^, which are explored in the present study. The objectives of this study were to: (1) determine mean breed BW in young adult dogs of different breeds and sex in Sweden, and (2) study changes in breed BW over a 10-year period in young adult male and female dogs.

## Materials and methods

For this observational study, a data file with hip radiographic screening results on dogs born during the years 2005–2015 was acquired from SKC in October 2018. The file held screening data from 2005 and onwards and contained information on the individual dogs, including registration number, breed, sex, date of birth, screening date and BW at time of screening.

### Data management

Cleaning of the data file was performed as follows: individuals without a Swedish registration number or with results from a former grading system were excluded from the file. Screening posts performed prior to the officially accepted age (12 months, or 18 months for certain large breeds) were excluded as were individuals with missing BW observations. Individuals with biologically implausible registered BWs (i.e. biologically impossible or extremely unlikely BWs for individual dogs, based on our understanding of biologically possible BWs for each specific breed)^27^ were excluded.

Screening results from 2006 and 2017/2018 were excluded, because the relative proportion of younger and older individuals, respectively, was different from the rest of the material. If an individual dog had more than one screening result, all but the first registered result were excluded. Individuals with screening results at an age of 12–24 months, or 18–30 months for certain large breeds (Tables 1 and Table S1), in breeds with at least 15 observations per year during the 10 years of screening (2007–2016), were included in the statistical analyses.

**Table 1:** Body weight (kg) per breed and sex for dogs screened during 2007–2016.

Breed	Male dogs		Female dogs		P-value
	n	LS means (SE)	n	LS means (SE)	
Alaskan Malamute	176	36.2 (0.6)	167	1.1 (0.6)	0.140
American Staffordshire Terrier	570	28.3 (0.2)	637	233.5 (0.2)	< 0.001
Australian Kelpie	417	19.4 (0.2)	422	15.9 (0.2)	0.605
Australian Shepherd	916	23.4 (0.1)	951	19.4 (0.1)	< 0.001
Bearded Collie	352	23.4 (0.2)	373	19.5 (0.2)	< 0.001
Belgian Shepherd Dog/Groenendael	173	23.7 (0.4)	191	19.7 (0.4)	0.002
Belgian Shepherd Dog/Malinois	613	29.7 (0.3)	622	24.5 (0.3)	< 0.001
Belgian Shepherd Dog/Tervueren	325	25.6 (0.3)	331	20.4 (0.3)	0.036
Bernese Mountain Dog	1839	46.1 (0.3)	2082	40.1 (0.3)	< 0.001
Border Collie	1383	19.1 (0.1)	1448	15.4 (0.1)	< 0.001
Boxer	1228	31.4 (0.1)	1335	25.8 (0.1)	< 0.001
Briard	309	36.4 (0.4)	365	29.2 (0.4)	0.004
Brittany	101	17.2 (0.3)	128	13.9 (0.3)	0.038
Bullmastiff*	145	54.5 (0.7)	210	46 (0.6)	0.231
Cane Corso	281	47.7 (0.5)	378	38.8 (0.4)	< 0.001
Chow Chow	215	26.9 (0.6)	275	24.2 (0.5)	0.037
Cocker Spaniel	953	13.9 (0.1)	1342	11.9 (0.1)	< 0.001
Collie Rough	992	24.3 (0.2)	1084	20.5 (0.2)	< 0.001
Collie Smooth	174	26.1 (0.3)	174	20.5 (0.4)	0.131
Dalmatian	149	32.1 (0.4)	151	26 (0.4)	0.007
Danish-Swedish Farmdog	764	9.1 (0.1)	1003	7.2 (0.1)	< 0.001
Dobermann	429	37.7 (0.3)	516	30.7 (0.3)	0.002
Dogue De Bordeaux*	142	57.4 (1.0)	192	48.6 (0.9)	0.206
East Siberian Laika	196	28.5 (0.3)	240	22.7 (0.3)	0.001
English Pointer	126	23.8 (0.4)	179	19.4 (0.3)	< 0.001
English Springer Spaniel	824	22.7 (0.2)	1119	19.2 (0.1)	< 0.001
Eurasian	310	25.1 (0.2)	384	21.8 (0.2)	0.006
Finnish Hound	172	27.3 (0.3)	240	22.3 (0.3)	0.088
Finnish Lapphund	744	17.3 (0.1)	833	14.8 (0.1)	< 0.001
Flat Coated Retriever	2378	31.8 (0.1)	2332	26.8 (0.1)	< 0.001
German Shepherd Dog	5798	35.7 (0.1)	6537	29.3 (0.1)	< 0.001
German Shorthaired Pointing Dog	364	29.4 (0.2)	361	23.9 (0.2)	0.003
German Spaniel	890	22.9 (0.1)	962	18.6 (0.1)	< 0.001
German Wirehaired Pointer	257	30.1 (0.3)	310	24.8 (0.2)	0.001
Giant Schnauzer, Black	359	37.7 (0.3)	361	30.2 (0.3)	0.032
Golden Retriever	5199	32.2 (0.1)	5761	27.7 (0.1)	< 0.001
Gordon Setter	129	26.3 (0.5)	149	21.2 (0.5)	0.020
Great Dane*	183	64.8 (0.9)	215	56.3 (0.8)	0.808
Hamilton Hound	129	25.5 (0.4)	157	21.3 (0.3)	0.071
Hovawart	438	37 (0.2)	442	30.2 (0.2)	< 0.001
Icelandic Sheepdog	91	15.3 (0.3)	127	13.4 (0.3)	0.001
Irish Red Setter	545	28.8 (0.3)	567	23.6 (0.3)	< 0.001
Irish Softcoated Wheaten Terrier	257	17 (0.2)	297	14.9 (0.2)	0.001
Keeshond	78	17.6 (0.4)	110	14.7 (0.3)	0.389
Labrador Retriever	6222	31.1 (0.1)	6903	26.2 (0.1)	< 0.001
Lagotto Romagnolo	1120	15.6 (0.1)	1328	13.4 (0.1)	< 0.001
Landseer*	108	53.6 (0.9)	151	45.9 (0.9)	0.732
Leonberger*	633	55.7 (0.4)	727	45.9 (0.4)	0.015
Newfoundland*	191	58.8 (0.7)	313	50.1 (0.6)	0.125
Norwegian Elkhound, Grey	804	19.5 (0.1)	825	16.6 (0.1)	< 0.001
Nova Scotia Duck Tolling Retriever	1095	19.8 (0.1)	1070	16.5 (0.1)	< 0.001
Poodle, Standard	520	23.3 (0.2)	629	18.9 (0.2)	0.004
Portuguese Water Dog	528	23 (0.2)	472	18.5 (0.2)	< 0.001
Pumi	183	12.5 (0.2)	196	10 (0.2)	0.253
Rhodesian Ridgeback	974	41.9 (0.2)	1065	34.8 (0.2)	< 0.001
Rottweiler	2773	45.4 (0.2)	3221	37.7 (0.2)	< 0.001
Saint Bernhard Dog, Long-Haired*	129	69.2 (1.0)	188	60.7 (0.9)	0.226
Samoyed	526	23.7 (0.2)	571	19.9 (0.2)	< 0.001
Schapendoes	136	17.6 (0.3)	162	14 (0.3)	0.064
Shetland Sheepdog	281	8.5 (0.2)	370	7.4 (0.2)	0.047
Shiba Inu	163	11.4 (0.2)	210	9.1 (0.2)	0.016
Small Münsterlander	207	23 (0.2)	234	19 (0.2)	0.015
Spanish Water Dog	559	18.8 (0.2)	604	15.6 (0.2)	0.016
Stabyhoun	155	22.1 (0.3)	170	18.5 (0.3)	0.062
Staffordshire Bull Terrier	964	18.2 (0.1)	1145	15.2 (0.1)	< 0.001
Swedish Elkhound	1986	27.7 (0.1)	1898	23.3 (0.1)	< 0.001
Swedish Lapphund	164	16.9 (0.3)	149	14.7 (0.3)	0.577
Swedish Vallhund	209	12.9 (0.2)	207	11 (0.2)	0.002
Tibetan Terrier	221	11.6 (0.2)	240	9.5 (0.2)	0.021
Welsh Springer Spaniel	771	19.4 (0.2)	863	16.4 (0.2)	< 0.001
White Swiss Shepherd Dog	287	34.3 (0.3)	319	28.3 (0.3)	0.080
Working Kelpie	129	20.5 (0.3)	127	16.4 (0.3)	0.165

For official registration of hip status by the Swedish Kennel Club, dogs should be at least 12 months old (18 months in some giant breeds, marked with *) https://www.skk.se/globalassets/dokument/uppfodning/broschyrer/rontgen-av-leder-hos-hund-a55.pdf

Dogs were 12–24 months of age at screening, except for dogs in breeds marked with *, which were 18–30 months old. Body weights were estimated in a general linear model and are presented as least square means (LS means) with standard error (SE). The P-value indicates the association between sex and body weight. The level of significance was set at P < 0.05.

After cleaning of the original data file (consisting of 183 252 records on dogs of 304 breeds), and applying the inclusion criteria, 114 568 dogs (64%) with individual BW observations from 72 breeds were included. The fraction of dogs participating in screening, of the registered dogs in the included breeds, ranged from roughly 5 to 72%, and was above 30% in 54 of the 72 breeds^28^.

### Statistical analysis

Commercially available software (SAS 9.4, SAS Institute Inc., Cary, NC, USA) was used for data management and statistical analyses. General linear models, using PROC GLM, were used to evaluate BW at screening and the change in breed BW over time, with BW considered a continuous variable with potentially normal distribution.

The analyses were performed separately for each breed. Year of screening was treated as a continuous variable, coded 1–10, and the other variables in the models were sex (male/female), age at screening and the interaction between sex and age at screening. To allow for non-linear associations between BW and age at screening, we also included a centered and squared term of age at screening, and the corresponding interaction term, in the models. Possible interactions between sex and year of screening, indicating different trends by sex, were initially evaluated and, if significant, the effect of year of screening was estimated nested within sex. Least-square means (LSMeans), or marginal means, were used to illustrate the associations between BW and sex and were evaluated at a ‘mean term’ for age at screening (547 days old at screening) and year of screening (the 6th screening year). Relative change in breed BW (%) over the 10 years was calculated as ten times the estimated regression coefficients divided by the mean breed BW from the first screening year. As adjustment for multiple testing is not required or preferred in hypothesis-generating studies^29,30^, no adjustment was performed.

The residuals were inspected for signs of non-normal distribution and heteroscedasticity, but no deviations were found. Influential observations were identified using Cook’s distance, but no abnormalities were found (all D’s < 0.20). Statistical level of significance was set at P < 0.05 for all analyses.

### Conference presentation

Presented in part at the 31st European College of Veterinary Internal Medicine—Companion Animal Congress, Online, September 2021.

## Results

The number of individuals per breed are shown in Table S1. The sex distribution was equal in the majority of breeds, with slightly more female dogs than male dogs in total (53 and 47%, respectively). Three of the 72 breeds (4%), had > 10 000 BW observations, 30 breeds (42%), had > 1000 observations and 54 breeds (75%) had > 500 observations on BW. Sixty-five of the breeds included dogs screened at 12–24 months of age, and the remaining seven breeds included dogs screened at 18–30 months of age (Table S1).

### Mean bodyweight per sex

Mean BWs per breed and sex are presented in Table 1. The BW ± SE ranged from 7.2 ± 0.1 kg in female Danish Swedish farm dogs to 69.2 ± 1.0 kg in male long-haired Saint Bernhard dogs. A significant breed BW difference between sexes was present in 54 (75%) of the breeds, indicated in the table by the P-value from estimates of the effect of sex on BW, with male dogs being more heavy in all breeds.

### Change in bodyweight over time

In Table S2, the estimates of the effect of year on BW are shown for each breed. A significant change in breed BW over the 10 years was present in 33 (46%) of the 72 breeds (Table 2). Of these, five breeds showed an increase in BW over time, with no difference between sexes (estimates in kg/year ranging from 0.077 in Bernese Mountain Dogs to 0.313 in Gordon Setters), and 22 breeds showed a decrease in BW over time, with no difference between sexes (estimates (kg/year) ranging from − 0.042 in Golden Retrievers to − 0.223 in Newfoundlands).

**Table 2:** Regression coefficients for the association between year of screening and body weight in breeds with a significant change during the screening years, 2007–2016.

Breed	Estimate	SE	P-value
American Staffordshire Terrier	− 0.220	0.032	< 0.001
Australian Kelpie	− 0.065	0.028	0.018
Australian Shepherd	− 0.134	0.024	< 0.001
Belgian Shepherd Dog/Groenendael	0.126	0.055	0.023
Belgian Shepherd Dog/Malinois, male dogs	0.165	0.048	0.001
Belgian Shepherd Dog/Malinois, female dogs	− 0.094	0.047	0.047
Bernese Mountain Dog	0.077	0.027	0.005
Border Collie	− 0.109	0.013	< 0.001
Boxer, male dogs	− 0.138	0.031	< 0.001
Bullmastiff	0.245	0.105	0.020
Chow Chow	− 0.162	0.051	0.002
Collie Rough	− 0.052	0.025	0.041
Collie Smooth	− 0.110	0.052	0.037
Dobermann	− 0.145	0.045	0.001
English Springer Spaniel	− 0.061	0.021	0.003
Finnish Lapphund	− 0.069	0.023	0.002
German Shepherd Dog	− 0.056	0.011	< 0.001
German Wirehaired Pointer	0.119	0.046	0.010
Golden Retriever	− 0.042	0.012	0.001
Gordon Setter	0.313	0.080	< 0.001
Irish Red Setter	− 0.136	0.044	0.002
Labrador Retriever	− 0.178	0.013	< 0.001
Lagotto Romagnolo, male dogs	− 0.246	0.022	< 0.001
Lagotto Romagnolo, female dogs	− 0.145	0.020	< 0.001
Newfoundland	− 0.223	0.108	0.040
Norwegian Elkhound, Grey	− 0.076	0.019	< 0.001
Rottweiler	− 0.138	0.021	< 0.001
Samoyed	− 0.077	0.034	0.024
Shetland Sheepdog	− 0.058	0.029	0.048
Shiba Inu, male dogs	0.089	0.043	0.038
Shiba Inu, female dogs	− 0.084	0.036	0.021
Spanish Water dog, male dogs	− 0.154	0.037	< 0.001
Staffordshire Bull Terrier	− 0.116	0.015	< 0.001
Swedish Elkhound	− 0.137	0.017	< 0.001
Swedish Lapphund	− 0.098	0.045	0.031
White Swiss Shepherd dog, female dogs	− 0.286	0.073	< 0.001

Results are presented as estimates in kg/year from a general linear model, with standard error (SE), for each breed. For breeds with a significant interaction between sex and year of screening, the estimates are given per sex. The level of significance was set at P < 0.05.

Interaction between sex and year was present in six breeds with a change in BW, and, therefore, estimates were established for each sex in these breeds (Table 2). In Lagotto Romagnolos, BW had decreased in both sexes, but with a larger reduction in male (− 0.246 kg/year) than in female (− 0.145 kg/year) dogs. In Boxers and Spanish Waterdogs, only male dogs showed a decrease in BW (− 0.138 and − 0.154 kg/year respectively), while in White Swiss Shepherd Dog, only female dogs showed a decrease in BW (− 0.286 kg/year). In Shiba Inus and Belgian Shepherd Dogs (Malinois), male dogs showed an increase in BW (0.089 and 0.165 kg/year respectively), while female dogs showed a decrease (− 0.084 and − 0.094 kg/year, respectively).

**Figure 1:**
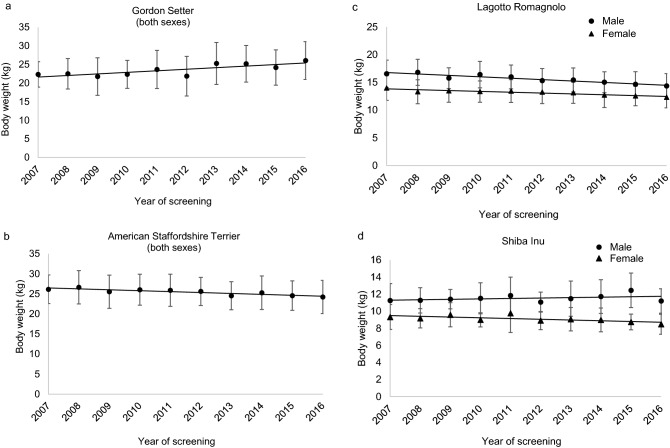
(**a-d**) Observed mean body weight (BW) ± standard deviation in selected breeds with significant change in BW during the study period (2007–2016). Linear regression lines based on the observed mean values have been applied to the graphs.

Relative and numerical changes in breed BW over the 10 years are shown in Tables 3 and 4. In breeds with no difference between sexes, Gordon Setters showed the largest relative and numerical increase in BW during the 10 years, 14% (3.1 kg), and American Staffordshire terriers the greatest decrease; 8% (− 2.2 kg) (Table 3, Fig. 1a,b). In breeds with differences between sexes, the change in BW differed both in direction and magnitude (Table 4). By way of example, Lagotto Romagnolos showed a decrease of 15% (− 2.5 kg) in male and 10% (− 1.4 kg) in female dogs (Fig. 1c), while Shiba Inu male dogs showed an increase of 8% (0.9 kg) and female dogs a decrease of 9% (− 0.8 kg) (Fig. 1d).

**Table 3:** Ten-year change in body weight in breeds without significant sex difference (screening years 2007–2016), based on estimates in a general linear model and presented in % and kg.

Breed	Change in body weight	
	%	kg
Gordon Setter	14.0	3.1
Belgian Shepherd Dog/Groenendael	5.9	1.3
Bullmastiff	5.1	2.5
German Wirehaired Pointer	4.7	1.2
Bernese Mountain Dog	1.9	0.8
American Staffordshire Terrier	8.4	− 2.2
Shetland Sheepdog	7.1	− 0.6
Staffordshire Bull Terrier	6.9	− 1.2
Australian Shepherd	6.3	− 1.3
Chow Chow	6.3	− 1.6
Border Collie	6.2	− 1.1
Swedish Lapphund	6.1	− 1.0
Labrador Retriever	6.0	− 1.8
Swedish Elkhound	5.3	− 1.4
Irish Red Setter	5.1	− 1.4
Collie Smooth	4.7	− 1.1
Dobermann	4.3	− 1.5
Finnish Lapphund	4.2	− 0.7
Norwegian Elkhound, Grey	4.1	− 0.8
Newfoundland	4.0	− 2.2
Australian Kelpie	3.7	− 0.7
Samoyed	3.4	− 0.8
Rottweiler	3.4	− 1.4
English Springer Spaniel	2.9	− 0.6
Collie Rough	2.3	− 0.5
German Shepherd Dog	1.7	− 0.6
Golden Retriever	1.4	− 0.4

Breeds with an increase in BW are presented first followed by breeds with a decrease in BW. Breeds are sorted in order of percentage change within the two groups.

**Table 4:** Ten-year change in body weight in breeds with interaction between sex and year of screening (screening years 2007–2016), based on estimates in a general linear model and presented in % and kg.

Breed	Change in body weight			
	Male dogs		Female dogs	
	%	kg	%	kg
Lagotto Romagnolo	14.9	− 2.5	10.4	− 1.4
Spanish Water Dog	8.0	− 1.5	0	0
Shiba Inu	7.9	0.9	9.0	− 0.8
Belgian Shepherd Dog/malinois	6.1	1.7	3.9	− 0.9
Boxer	4.4	− 1.4	0	0
White Swiss Shepherd Dog	0	0	9.5	− 2.9

Breeds are sorted according to percentage change in male dogs. Note that a negative figure in kg denotes a decrease in body weight.

## Discussion

In this large observational register-based study, we have estimated BW in young adult dogs of 72 different breeds in Sweden. In 33 of the breeds, a change in breed BW during the 10-year period was noticed, and, although not a prominent change for most, it was greater than 10% in some breeds.

The study population consisted of young adult dogs; 12–24 months old for most breeds and 18–30 months old for certain larger breeds. Adult canine BW at maturity depends on both genetic and environmental effects, and is reached at different ages. Factors including nutritional status and body condition, as well as breed, size and sex, have been associated with growth, time to maturity and adult BW^2–5,31^. The lowest age for an official hip screening result is 12 or 18 months. The age limit is set in agreement between international kennel club organizations and is based on age at maturity for each breed, with larger breeds reaching maturity later. In Sweden, dogs are traditionally examined at the allowed age for an official result or shortly thereafter, with results publically available in a database^28^. The inclusion criteria for age in the study were set with the intention to include dogs from young adult age at an age interval when the majority of dogs are screened.

The sex distribution was equal in most breeds in this study, which reflects the sex distribution in all dogs registered in SKC, as well as in the Swedish dog population^32^. The BW differed significantly between sexes in 54 of the 72 breeds, with male dogs being heavier. Studies have shown slower growth in male dogs of several breeds, but with higher BW at the end of the growth period than female dogs of the same breed^3,5,31^. The non-significant difference between sexes in some breeds in this study might depend on some participating individuals, especially in the larger breeds, not having reached fully adult BW at the time of screening^3,4^. Potentially, varying breeding priorities regarding sex characteristics between different breeds, might also affect the outcome. Included breeds were mostly medium to large sized breeds, which generally have a higher prevalence of hip dysplasia^33,34^ and therefore a larger number of screenings, compared to smaller sized breeds. Even if the screening program is available for all breeds, screening is a requirement before registration of offspring in certain breeds, while specific screening results are needed before registration in other breeds^26^. Therefore, the population coming to screening likely differs between breeds. There is a long tradition to screen dogs for HD in Sweden^26^, which reflects the individual dogs participating in the program. Being a tool for selection of breeding stock, many presumable breeding dogs attend the program. However, the majority of dogs attending the screening are not breeding animals^28^, but offspring participating for parental breeding evaluation or pet dogs participating for joint health evaluation, since the screening result indicates risk of future clinical signs and can thereby be of value for the individual owner^15^.

Adult BW is affected not only by body size, but also by body composition i.e. the proportion of lean mass and fat. Since such information was not available in the data set, it is not known how the BW reflects size or body composition of included dogs. Thus, despite the extensive study population it is difficult to evaluate whether dogs in this study population are representative, i.e. lighter or heavier than dogs in general, in Sweden as well as globally. The BWs given in the breed standards might not be valid references for comparison. They are not only commonly given as ranges or maximums, but might also be based on estimations and assumptions made decades ago, when the variation within breeds was greater than today.

Approximately 45% of the studied breeds showed a change in BW during the 10-year period. The change could be due to changes in body fat mass and/or in size. Regarding the health risks associated with high BW, due to excessive body fat or large size, the decrease in BW observed in several breeds, might imply a reduced risk of known health disadvantages, including orthopedic disorders^10,11,13–16,35^. The breeds with the most prominent decreases in BW, Lagotto Romagnolo, American Staffordshire Terriers, Staffordshire Bullterriers and Spanish Waterdogs, are primarily companion dogs in Sweden, but also to a great extent attend shows and competitions. These four breeds had a screening hip dysplasia prevalence of 30–65% during the studied years^28^, and the decrease in their breed BW could improve that situation. They are all popular breeds, and an increasing popularity is reflected in extensively rising numbers of registered individuals during the study years for Staffordshire Bullterriers and Spanish Waterdogs^28^. The rising popularity might have affected the population coming to screening and thus, the breed BW.

The five breeds that had a similar increase in BW in both sexes, i.e. Gordon Setter, Belgian Shepherd/Groenendael, Bullmastiff, German Wirehaired Pointer and Bernese Mountain Dog, were larger breeds of different types and uses. The largest increase was found in Gordon Setters. If this increase is due to an increased fat mass, it would reflect a one-step change in the semi-quantitative 9-point scale for body condition score (BCS)^36^. The Gordon Setter is a bird hunting dog, which has been bred into a field type and a show type. The show type is heavier than the field type, and a possible preference for the show type in later years may explain some of the observed change in breed BW in Gordon Setters. The Bullmastiff, one of the heaviest breeds in our study with a mean breed BW of 54.5 kg in male, and 46.0 kg in female dogs, respectively, also showed an increase in BW. The reported screening prevalence of hip dysplasia in the breed was > 50% during the study years in Sweden^28^. With BW being a risk factor for development of osteoarthritis in large-size breeds with hip dysplasia^14,37^, and the known risk of increased morbidity and mortality in these dogs^15^, the increase in breed BW in the Bullmastiff, might have negative impact on their overall health and lifespan, regardless of the underlying cause.

Internationally reported prevalence of overweight and obesity in dogs is high, and seems to be increasing^18–22,24,38^. Concerns for a general trend towards heavier show dogs, both older and younger, are being discussed^23,38^, even though recommended BW and height in breed standards have not changed for the last decades. In consistency with the internationally published figures, a recent study on Swedish show dogs found 32% of the dogs being overweight^23^. The observed decrease in mean BW in several breeds in our study might as previously mentioned, relate to either changes in size or body condition. One potential explanation for the decrease could be a change in dog owner’s view of normal body condition in young, active dogs of certain breeds during the 10-year period. It is, however, important to be aware that the study population itself and possible changes in its composition, might impact the results. Data was collected in conjunction with a screening program and the dogs were presumably healthy, young, adult dogs, in which overweight due to excess of body fat tends to be less common than in middle-aged and older dogs^18,19,21,25,39^. The assumption of dogs being presumably healthy is based on the intention of the screening program, i.e. being a tool for breeding evaluation and for evaluation of future joint health. The number of neutered dogs in the study population is most probably low, based on the participating potential breeding animals and their offspring, and the tradition in Sweden not to neuter young dogs extensively without medical reason. The effect of neutering on BW in this study could therefore be considered to be low, in consistency with a previous study on BW in young dogs^3^. Changes in breed popularity and usage are factors that might affect our results. Certain breeds containing subgroups with different phenotypes were included as one in the dataset. The Labrador Retriever, for example, showed a decrease in breed BW, despite being a breed with reported high prevalence of overweight^25,40,41^, and an identified gene mutation associated with adiposity and food motivation^42^. The division into two almost different breed types with the leaner hunting type increasing in popularity, might account for the unexpected decrease in BW in this breed.

There are some limitations to this study. The study population consists of dogs registered in SKC and participating in a screening program, which might lead to selection bias, and affect the representativeness for the general dog population in Sweden. The breed representation does not cover all breeds due to inclusion criteria on number of observations, and smaller sized breeds are therefore underrepresented. Our criteria also exclude dogs older than 2.5 years of age. However, around 70% of the total dog population in Sweden are registered in SKC, and the proportion of dogs participating in screening was above 30% in 54 of the included breeds, which is in agreement with the fraction screened in many breeds in SKC^26^. Thus, based on the above, and considering the large data set, we assess that our study population represents the included breeds and age groups relatively well. However, the rules regarding registration of dogs in SKC could also affect the results. For example, breed variations and subgroups are present in some dog breeds, based on e.g. size or coat color and texture, and therefore registered separately in SKC, while other breeds with obvious division into phenotypically different subgroups register as one. Rules for registration in SKC and for screening might have changed in certain breeds during the study period, which could affect the results. Lacking information about the body condition of the dogs, it is unknown whether the population described can be considered ideal weight or not, and what observed breed BW changes depend on. Finally, being an observational study, results of changes in BW over time need to be interpreted with caution, especially for breeds in which changes were small, and should be confirmed in further studies.

In summary, this study provides BW information for a large number of breeds, based on data from young adult dogs in Sweden, mandatorily registered in conjunction with a hip screening program. These breed BWs could proposedly be used as a guide, complementing other sources on adult breed BW. Over the 10-year period, a change in BW was present in around 45% of the breeds with a change greater than 10% in some. Lacking details about body condition, and thereby knowledge of what constitutes the BW, this could be a consequence of changes either in breed-related size or in body fat mass. Still, the change in BW in certain breeds might have an impact on the overall health within those breeds. Future studies with simultaneous evaluation of BW and body condition over time are warranted and might further elucidate this matter.

## Supplementary Information

Supplementary Tables.

## Data Availability

The data that support the findings of this study are available from the Swedish Kennel Club but restrictions apply to the availability of these data, which were used under approval for the current study, and so are not publicly available. Data are however available from the corresponding author (linda.andersson@slu.se) upon reasonable request and with permission from the Swedish Kennel Club.
