# Supplementary material for: Exploration of body weight in 115 000 young adult dogs of 72 breeds
Source: Sci Rep. 2023 Jan 9;13:443. doi: 10.1038/s41598-022-27055-4 (PMC9829868; doi:10.1038/s41598-022-27055-4)
Supplement: Supplementary file 1 — Supplementary Tables. [file 41598_2022_27055_MOESM1_ESM.pdf]

Exploration of body weight in 115 000 young adult dogs of 72 breeds

Andersson, L.,[a*] Emanuelson, U.,[b] Ringmark, S.,[a] Bjørnvad, C.R.,[c] Hedhammar, Å.,[b] Höglund, K.[a]

a Department of Anatomy, Physiology and Biochemistry, Swedish University of Agricultural Sciences, Box 7054, 75007 Uppsala, Sweden

b Department of Clinical Sciences, Swedish University of Agricultural Sciences, Box 7054, 75007 Uppsala, Sweden

c Department of Veterinary Clinical Sciences, Faculty of Health and Medical Sciences, University of Copenhagen, Dyrlægevej16, 1870 Frederiksberg C, Denmark

[*]Corresponding author linda.andersson@slu.se

Supplementary tables:

Table S1. Number (n) of observations on body weight per breed and sex in 114 568 dogs. Dogs were 12-24 months of age at screening, except for dogs in breeds marked with *, which were 18-30 months old.

	Total	Male dogs	Female dogs
**Breed**	**n**	**%**	**%**
Alaskan Malamute	343	51.3	48.7
American Staffordshire Terrier	1207	47.2	52.8
Australian Kelpie	839	49.7	50.3
Australian Shepherd	1867	49.1	50.9
Bearded Collie	725	48.6	51.4
Belgian Shepherd Dog/ Groenendael	364	47.5	52.5
Belgian Shepherd Dog/ Malinois	1235	49.6	50.4
Belgian Shepherd Dog/ Tervueren	656	49.5	50.5
Bernese Mountain Dog	3921	46.9	53.1
Border Collie	2831	48.9	51.1
Boxer	2563	47.9	52.1
Briard	674	45.8	54.2
Brittany	229	44.1	55.9
Bullmastiff*	355	40.8	59.2
Cane Corso	659	42.6	57.4

Chow Chow	490	43.9	56.1
Cocker Spaniel	2295	41.5	58.5
Collie Rough	2076	47.8	52.2
Collie Smooth	348	50.0	50.0
Dalmatian	300	49.7	50.3
Danish-Swedish Farmdog	1767	43.2	56.8
Dobermann	945	45.4	54.6
Dogue De Bordeaux*	334	42.5	57.5
East Siberian Laika	436	45.0	55.0
English Pointer	305	41.3	58.7
English Springer Spaniel	1943	42.4	57.6
Eurasian	694	44.7	55.3
Finnish Hound	412	41.7	58.3
Finnish Lapphund	1577	47.2	52.8
Flat Coated Retriever	4710	50.5	49.5
German Shepherd Dog	12335	47.0	53.0
German Shorthaired Pointing Dog	725	50.2	49.8
German Spaniel	1852	48.1	51.9
German Wirehaired Pointer	567	45.3	54.7
Giant Schnauzer, Black	720	49.9	50.1
Golden Retriever	10960	47.4	52.6
Gordon Setter	278	46.4	53.6
Great Dane*	398	46.0	54.0
Hamilton Hound	286	45.1	54.9
Hovawart	880	49.8	50.2
Icelandic Sheepdog	218	41.7	58.3
Irish Red Setter	1112	49.0	51.0
Irish Softcoated Wheaten Terrier	554	46.4	53.6
Keeshond	188	41.5	58.5
Labrador Retriever	13125	47.4	52.6
Lagotto Romagnolo	2448	45.8	54.2
Landseer*	259	41.7	58.3
Leonberger*	1360	46.5	53.5
Newfoundland*	504	37.9	62.1
Norwegian Elkhound, Grey	1629	49.4	50.6
Nova Scotia Duck Tolling Retriever	2165	50.6	49.4
Poodle, Standard	1149	45.3	54.7
Portuguese Water Dog	1000	52.8	47.2
Pumi	379	48.3	51.7
Rhodesian Ridgeback	2039	47.8	52.2
Rottweiler	5994	46.3	53.7

Saint Bernhard Dog, Long-Haired*	317	40.7	59.3
Samoyed	1097	47.9	52.1
Schapendoes	298	45.6	54.4
Shetland Sheepdog	651	43.2	56.8
Shiba Inu	373	43.7	56.3
Small Münsterlander	441	46.9	53.1
Spanish Water Dog	1163	48.1	51.9
Stabyhoun	325	47.7	52.3
Staffordshire Bull Terrier	2109	45.7	54.3
Swedish Elkhound	3884	51.1	48.9
Swedish Lapphund	313	52.4	47.6
Swedish Vallhund	416	50.2	49.8
Tibetan Terrier	461	47.9	52.1
Welsh Springer Spaniel	1634	47.2	52.8
White Swiss Shepherd Dog	606	47.4	52.6
Working Kelpie	256	50.4	49.6
	**114568**	**47.3**	**52.7**

For official registration of hip status by the Swedish Kennel Club, dogs should be at least 12 months old (18 months in some giant breeds, marked with *) https://www.skk.se/globalassets/dokument/uppfodning/broschyrer/rontgen-av-leder-hos-hund-a55.pdf

Table S2. Regression coefficients for the association between year of screening and body weight (screening years 2007-2016) for all included breeds. Results are presented as estimates in kg/year from a general linear model, with standard error (SE), for each breed. For breeds with a significant interaction between sex and year of screening, the estimates are given per sex. The level of significance was set at P<0.05.

Breed	Estimate	SE	P- value
Alaskan Malamute	- 0.088	0.101	0.380
American Staffordshire Terrier	- 0.220	0.032	<0.001
Australian Kelpie	- 0.065	0.028	0.018
Australian Shepherd	- 0.134	0.024	<0.001
Bearded Collie	- 0.009	0.030	0.776
Belgian Shepherd Dog/ Groenendael	0.126	0.055	0.023
Belgian Shepherd Dog/ Malinois			
Male dogs	0.165	0.048	0.001
Female dogs	- 0.094	0.047	0.047
Belgian Shepherd Dog/ Tervueren	0.005	0.043	0.900
Bernese Mountain Dog	0.077	0.027	0.005
Border Collie	- 0.109	0.013	<0.001
Boxer			
Male dogs	- 0.138	0.031	<0.001
Female dogs	- 0.038	0.029	0.182
Briard	- 0.012	0.059	0.837
Brittany	- 0.049	0.051	0.335
Bullmastiff	0.245	0.105	0.020
Cane Corso	0.116	0.078	0.139
Chow Chow	- 0.162	0.051	0.002
Cocker Spaniel	0.001	0.011	0.954
Collie Rough	- 0.052	0.025	0.041
Collie Smooth	- 0.110	0.052	0.037
Dalmatian	- 0.031	0.062	0.614
Danish-Swedish Farmdog	- 0.005	0.012	0.700
Dobermann	- 0.145	0.045	0.001
Dogue De Bordeaux	0.247	0.133	0.064
East Siberian Laika	0.010	0.051	0.845
English Pointer	0.035	0.049	0.478
English Springer Spaniel	- 0.061	0.021	0.003
Eurasian	0.000	0.040	0.992
Finnish Hound	- 0.065	0.050	0.195
Finnish Lapphund	- 0.069	0.023	0.002
Flat Coated Retriever	- 0.034	0.017	0.052
German Shepherd Dog	- 0.056	0.011	<0.001

German Shorthaired Pointing Dog	- 0.067	0.036	0.061
German Spaniel	- 0.010	0.019	0.585
German Wirehaired Pointer	0.119	0.046	0.010
Giant Schnauzer, Black	- 0.079	0.052	0.128
Golden Retriever	- 0.042	0.012	0.001
Gordon Setter	0.313	0.080	<0.001
Great Dane	0.055	0.107	0.607
Hamilton Hound	0.034	0.054	0.528
Hovawart	- 0.082	0.044	0.062
Icelandic Sheepdog	0.012	0.046	0.800
Irish Red Setter	- 0.136	0.044	0.002
Irish Softcoated Wheaten Terrier	0.026	0.029	0.358
Keeshond	0.017	0.056	0.754
Labrador Retriever	- 0.178	0.013	<0.001
Lagotto Romagnolo			
Male dogs	- 0.246	0.022	<0.001
Female dogs	- 0.145	0.020	<0.001
Landseer	0.148	0.138	0.286
Leonberger	0.013	0.060	0.823
Newfoundland	- 0.223	0.108	0.040
Norwegian Elkhound, Grey	- 0.076	0.019	<0.001
Nova Scotia Duck Tolling Retriever	- 0.020	0.019	0.295
Poodle, Standard	0.003	0.026	0.911
Portuguese Water Dog	- 0.020	0.032	0.534
Pumi	- 0.025	0.037	0.503
Rhodesian Ridgeback	- 0.043	0.033	0.196
Rottweiler	- 0.138	0.021	<0.001
Saint Bernhard Dog, Long-Haired	- 0.050	0.153	0.745
Samoyed	- 0.077	0.034	0.024
Schapendoes	0.024	0.044	0.589
Shetland Sheepdog	- 0.058	0.029	0.048
Shiba Inu			
Male dogs	0.089	0.043	0.038
Female dogs	- 0.084	0.036	0.021
Small Münsterlander	- 0.054	0.040	0.176
Spanish Water Dog			
Male dogs	- 0.154	0.037	<0.001
Female dogs	- 0.028	0.035	0.424
Stabyhoun	- 0.018	0.052	0.731
Staffordshire Bull Terrier	- 0.116	0.015	<0.001
Swedish Elkhound	- 0.137	0.017	<0.001
Swedish Lapphund	- 0.098	0.045	0.031

Swedish Vallhund	0.006	0.021	0.781
Tibetan Terrier	0.017	0.028	0.545
Welsh Springer Spaniel	0.014	0.018	0.440
White Swiss Shepherd Dog			
Male dogs	- 0.046	0.074	0.536
Female dogs	- 0.286	0.073	<0.001
Working Kelpie	- 0.040	0.052	0.436
